# The study of Fe_3_O_4_@SiO_2_-NH_2_ nano-magnetic composite modified by glutaraldehyde to immobilized penicillin G acylase

**DOI:** 10.3906/kim-2103-60

**Published:** 2021-09-14

**Authors:** Monier Alhadi Abdelrahman MOHAMMED, Zhenbin CHEN, Ke LI, Boyuan ZHANG

**Affiliations:** 1 College of Materials Science and Engineering, Lanzhou University of Technology, Lanzhou, People’s Republic of China; 2 State Key Laboratory of Gansu Advanced Non-ferrous Metal Materials, Lanzhou University of Technology, Lanzhou, People’s Republic of China; 3 Department of Chemistry, Faculty of Science, University of Kordofan, Alobaied, Sudan

**Keywords:** Penicillin G acylase, immobilization, nano-magnetic composite, activity, reusability

## Abstract

Preparation of biocatalyst dependent on immobilized penicillin G acylase (PGA) was of substantial importance for proteomic research, organic synthesis, and industrial applications. Herein, we developed an easy method for nano-magnetic composite to immobilize PGA. Fe_3_O_4_ nano-magnetic particles were co-precipitated with Fe^3+^ and Fe^2+^ in an ammonia solution (NH_3_) and treated with silicon dioxide (SiO_2_), which was developed using the sol-gel process. Thereafter, 3-aminopropyltriethoxysilane (APTES) was used to modify the silica-coated Fe_3_O_4_, which would result in the attachment of the primary amine groups to the particle surface. After that, the attachment of primary amine group was reacted with glutaraldehyde (Glu) to immobilize PGA; the products related to each step were confirmed by X-ray diffraction (XRD), Fourier-transform infrared spectroscopy (FTIR), vibration sample magnetometer (VSM), and scanning electron microscope-energy spectroscopy of dispersive x-rays (SEM-EDS). Condition investigation results revealed that the suitable pH value, reaction time, and immobilization temperature were 8.0, 6 h, and 40 °C, respectively, under optimal conditions. Enzyme loading capacity (ELC), enzyme activity (EA), and enzyme activity retention ratio (EAR) of PGA were 9198 U, 14602 U/g, and 87.7% respectively. Reusability findings showed that the immobilization PGA preserved 79% of its activity after 11 cycles of repeating.

## 1. Introduction

Magnetic nanomaterials had gained tremendous interest in recent years leading to their remarkable magnetic propertiesand had been practically used in the biology and biomedicine fields [1–4]. Superparamagnetic magnetite (Fe_3_O_4_), has been developed as appropriate candidates for different biomedical technologies, such as magnetic resonance imaging [5–7], treatment of hyperthermia [8, 9], targeted delivery of drug [10–13], labeling, cells sorting [14], and isolation of biologicalproducts [1,13,15], based on super magnetic properties. A substantial number of magnetic nanoparticles, which werecommonly made up of Fe_3_O_4_ magnetic nanoparticles and a synthetically modifiable shell, such as, SiO_2_ [16], Au [17], LDH [18], polyglycidyl methacrylate [19], polystyrene [20], etc. had been synthesized. Among them, SiO_2_ was widelyregarded as the best shell component because of its advantages in the maintenance of magnetic properties for the core ofFe_3_O_4_, trustworthy chemical stability, biocompatibility, and surface modification versatility [21, 22]. Furthermore, it couldprovide binding sites or organic polymers, bioactive molecules, and radicals because many silanol groups are distributedon the surface [23].

With the development of the study, Fe_3_O_4_@SiO_2_ used as an enzyme immobilization carrier received particular attention leading to specific magnetic susceptibility, lower toxicity, and synthetic-modifying surface [24–34]. PGA played an important biocatalyst and catalyzed the reaction of penicillin G (PG) potassium salt in semi-synthetic antibiotics to form 6-aminopenicillinc acid (6-APA) and other semi-synthetic penicillins [35–38] because the reaction under the catalytic ofPGA would take place at mild and environmentally friendly conditions at high production. In the application process, theeffective recovery and recycle of PGA was always a necessary requirement economically. However, free PGA was limitedseriously in industrial applications due to inactivation, poor stabilization, and stress of recycling [39]. The maximumabsorbance potential of mesoporous homogenizing materials to PGA could increase the catalytic operation, so this actionwas adopted to immobilize PGA. However, leaking of some PGA throughout a sustained reaction would influence theload capacity resulting from poor physical adsorption interaction between carrier and PGA [40]. Besides, attaching PGAto the mesoporous carrier substrate indicated that it must endure a higher steric interference and propagation limitation; furthermore, a large number of interest sites could not be filled, resulting in the reduced retention rate [41].

Many enzyme immobilization techniques have been used, such as adsorption, covalent binding, entrapment, and cross-linking. Adsorption creates a mild acting force between carriers and enzymes, causing the enzyme to slip off readily. The embedding technique leads to a significant diffusion resistance and enzyme deactivation. The method of cross-linking affects the active structure of the enzyme. While covalent binding was extensively used in the current enzyme industry because enzymes bond effectively with carriers and do not easily fall off. Whereas, if PGA was covalently immobilized on the magnetic nanoparticles’ surface [42, 43], it could eliminate leakage of PGA at a high degree. Besides, the recovery would also be simplified to a certain degree. However, Fe_3_O_4_ magnetic nanoparticles could easily be oxidized under the atmosphere, which means that recovery improvement would be confined to an obvious degree. Therefore, the development and planning of a unique type of carrier capable of handling stable load capacity, the quick response speed, and higher PGA recovery still had a high impact and challenges. At the same time, naked magnetic nanomaterials have highly reduced reactivity, resulting in instability and aggregation [44]. For these considerations, magnetic nanomaterials were frequently coated with silica to improve their stability and biocompatibility, and the silica can supply the hydroxy for surface modification. To accelerate the immobilization of enzymes, glutaraldehyde (Glu) is a strong cross-linker that can be connected to amino groups of PGA, maintaining the functionality of (Glu) cross-linked PGA active sites are not damaged [45,46]. (Glu) has been used to accomplish covalent immobilization of PGA precisely due to the efficiency of (Glu) chemistry.

In this work, we prepared Fe_3_O_4_@SiO_2_-NH_2_ nano-magnetic composite via a Stöber process and modified it with Glu (Fe_3_O_4_@SiO_2_-NH_2_-Glu) for the covalent immobilization of PGA. The properties of the immobilized PGA were investigated, and a practical immobilization approach for enzyme onto Fe3O4@SiO2-NH2-Glu) nano-magnetic composite was established with immobilized PGA as a model.

## 2. Materials and methods

### 2.1. Materials

FeCl_2_.4H_2_O, FeCl_3_.6H_2_O, and ethanol (C_2_H_5_OH) were analytical grade and purchased from Tianjin Baishi Chemical Co. Ltd. China. Tetraethylorthosilicate (TEOS), 3-aminopropyltriethoxysilane (APTES) were analytical grade and supplied by Shanghai Macklin Biochemical Co. Ltd. China. Aqueous ammonia (NH_3_) was obtained from Sichuan Ailing Chemical Co, Ltd. Phosphoric acid (H_3_PO_4_), potassium dihydrogen phosphate (KH_2_PO_4_), and dipotassium hydrogen phosphate (K_2_HPO_4_.3H_2_O) were analytical grade and obtained from Tianjin Guangfu Technology Co., LTD., China. Penicillin G acylase (PGA, the original free enzyme activity was 16900 U/g), 6-aminopenicillinic acid (6-APA, AR), Paradimethylaminobenzaldehyde (PDAB, AR), Penicillin G potassium (PG, AR), glutaraldehyde (C_5_H8O_2_, AR), hydroxylamine hydrochloride (HONH_3_Cl, AR) were purchased from Hubei Blue Sky Pharmaceutical Co. LTD. Distilled water was provided by our laboratory.

### 2.2. Methods

#### 2.2.1. Relevant solutions preparation

##### 2.2.1.1. Preparation of glutaraldehyde solution

A total of 0.200 g of 50 % glutaraldehyde solution was diluted with distilled water to volumetric flask 100.000 mL to produce a solution with a concentration of 1.000 g/L. After that, the glutaraldehyde solution was utilized immediately in the following process.

##### 2.2.1.2. Preparation of hydroxylamine hydrochloride

A total of 0.200 g hydroxylamine hydrochloride kept away from damp was transferred to a beaker 100.000 mL which had been loaded with an adequate amount of deionized water and stirred to dissolve completely. A 100.000 mL of the volumetric flask was loaded with the solution. The beaker was washed with distilled water repeatedly three times, and transferred the volumetric flask. Finally, the solution of 2.000 g/L hydroxylamine hydrochloride was obtained.

##### 2.2.1.3. Preparation of phosphate buffer solution

A total of 8.260 g of Potassium dihydrogen phosphate and 0.550 g of dipotassium hydrogen phosphate were placed into a beaker which had filled with 1000 mL of distilled water, and then pH value was adjusted using a 0.001 mol/L of KOH and H3PO4, pH = 7.8. During this process, a pH model meter was used to monitor, and eventually, 0.040 mol/L, phosphate buffer solution with pH = 7.8, was obtained [35].

##### 2.2.1.4. 6-APA Preparation

1.000 g of 6-amino penicillinc acid was dissolved into the above-prepared solution of phosphate buffer, the mixture was added to the volumetric flask (100 mL). Washing the beaker with the prepared phosphate buffer solution three times, and the solution was also transferred to the volumetric flask. Finally, a typical solution of 10.000 g/L of 6-APA was obtained [45].

##### 2.2.1.5. Preparation of PDAB solution

A total of 4.000 g of PDAB was dissolved with an ethanol solution of H_3_PO_4_ (the volume ratio of ethanol to H_3_PO_4_ was 93:7), and the mixture was placed into a 100.000 mL volumetric flask. Finally, 40.00 g/L standard ethanol of PDAB was prepared. After that, this solution was immediately used.

##### 2.2.1.6. Preparation of PGA solution

A total of 2.500 g of the free PGA stock solution was pipetted into a 100.000 mL volumetric flask, a phosphate buffer solution at pH = 7.8 was adopted to bring a volumetric flask to the scale, and a PGA solution at a concentration of 25.000 g/L was obtained.

##### 2.2.1.7. Preparation of penicillin G potassium solution

A total of 5.500 g of penicillin G potassium was dissolved into an appropriate amount of above-prepared phosphate buffer solution, completely stirred with a glass rod, and then transferred into 100.000 mL of volumetric flask. Finally, the volumetric flask was brought to the scale using a buffer solution, and the obtained 55.000 g/L of PG solution was preserved in a refrigerator at 4 °C for use in the following process.

#### 2.2.2. Preparation of Fe_3_O_4_ nano-magnetic particles

A simple chemical co-precipitation method was used to prepare Fe_3_O_4_ nano-magnetic particles [47]. A total of 7.570 g of FeCl_3_.6H_2_O and then 4.450 g of FeCl_2_.4H_2_O (molar ratio 2:1) were soluble under nitrogen control in 250 mL of distilled water at ambient temperature. Then, the temperature was raised to 80 °C, and 20 mL aqueous ammonia (25%) was introduced into the mixture by drops. The black reaction mixture precipitated was isolated using an external magnet after it had been stirred for 45 min and then washed three times with distilled water. Lastly, magnetic nanoparticles were completely dried overnight in a vacuum oven at 45 °C.

#### 2.2.3. Preparation of Fe_3_O_4_@SiO_2_ nano-magnetic particles

Updated stöber procedure had been used to coat Fe_3_O_4_ magnetic nanoparticles with SiO_2_ shells [48]. In short, Fe_3_O_4_ (1.000 g) freshly prepared and added in 200 mL ethanol, and 50 mL distilled water and sonicated with 5 mL of aqueous ammonia (25%) for 45 min. 3.500 mL TEOS was slowly mixed into the reaction solution while being mechanically stirred. The resultant distribution was mechanically stirred for 20 h at ambient temperature. Magnetic separation collected nano-magnetic particles and washed repeatedly three times using ethanol and distilled water, ultimately being dried in a 45°C vacuum laboratory oven.

#### 2.2.4. Activation of Fe_3_O_4_@SiO_2_ nano-magnetic particles-withNH_2_ group

The function of silica-coated superparamagnetic (Fe_3_O_4_@SiO_2_ nano-magnetic particles) had been performed using APTES. 1.000 g of Fe_3_O_4_@SiO_2_ nano-magnetic particles were dispersed into 50 mL of ethanol and sonicated for 45 min at room temperature. Afterwards, 2.00 mL APTES was added, and the solution was warmed to 70 °C for 20 h with vigorous stirring. The final (Fe_3_O_4_@SiO_2_-NH_2_) production was washed three times with ethanol and acetone using magnetic decantation and dried overnight under vacuum at 45 °C.

#### 2.2.5. Preparation of Fe_3_O_4_@SiO_2_-NH_2_-Glu nano-magnetic composite

A total of 0.500 g of Fe_3_O_4_@SiO_2_-NH_2_ was dispersed into 50.000 mL phosphate buffer solution at pH = 7.8, and 2.500 mL of 1.000 g/L glutaraldehyde solution was introduced. Then, the suspension was heated at 37 °C for 2 h. The glutaraldehyde-activated nanomagnetic composites (Fe_3_O_4_@SiO_2_-NH_2_-Glu) were isolated using magnetic decantation and washed carefully with sodium phosphate buffer solution.

#### 2.2.6. PGA immobilization onto Fe_3_O_4_@SiO_2_-NH_2_-Glu nano-magnetic composite

A total of 0.500 g of Fe_3_O_4_@SiO_2_-NH_2_-Glu dispersed into 10.000 mL of 25.000 g/L PGA solution was used to synthesize Fe_3_O_4_@SiO_2_-NH_2_-Glu nano-magnetic composites under stirring at 200 rpm at 37 °C for 24 h. The product magnetically was isolated and then washed with the above phosphate buffer solution, the final sample (Fe_3_O_4_@SiO_2_-NH_2_-Glu-PGA) was collected for the following use.

#### 2.2.7. Determination of free PGA Catalytic activity and immobilization PGA loading capacity

A total of 0.500 mL of 25.000 g/L PGA with concentration was added into 5.000 mL of 55.000 g/L PG solution and incubated for 5 min in a 37 °C oscillator. Following 0.500 mL from obtained solution was diluted three-fold using the phosphate buffer solution mentioned above. Then 0.500 mL of the dilution aforementioned above was added to a cuvette containing 3.500 mL PDAB solution with a concentration 40.000 g/L. After the reaction had been running for 3 min, the absorbance at 420 nm was determined three times with an ultraviolet spectrophotometer. Equations (1) and (2) were used to measure the catalytic function of the free PGA and immobilization PGA loading capacity, respectively.


(1) $${\text{EA}}_{\text{v}}=\frac{\text{C}\times \text{V}}{\text{V}0\times \text{t}}$$

Where EA_v_ referred to the catalytic activity of free PGA (U/mL); C referred to the 6-APA concentration (mM), which was calculated according the standard curve, V denoted the v the system volume (mL), V_0_ stood for the volume of the free PGA (mL), and t denoted to the reaction time (min).


(2) $$\text{ELC}=({\text{EA}}_{\text{v}0}-{\text{EA}}_{\text{vr}})\times \text{V}$$

ELC referred to the immobilization PGA loading capacity (U), EA_v0_ denoted the free PGA catalytic activity in original solution (U/mL), the value was 16 900 U/g. EA_vr_ referred the residual liquid activity of immobilization PGA (U/mL). V was reaction time volume (mL).

#### 2.2.8. The measure of immobilized PGA activity retention ratio

A total of 0.200 g of immobilization PGA was followed with 5.000 mL of 55.000 g/L PG solution and incubated for 5 min in a 37 °C oscillator. Then, 0.500 mL of the obtained solution was diluted three-fold using the phosphate buffer solution mentioned above. A total of 0.500 mL of the above solution was added to a cuvette containing 3.500 mL PDAB solution with a concentration 40.000 g/L. After the reaction had been running for 3 min, the absorption at 420 nm was measured three times using an ultraviolet spectrophotometer. Equations (3) and (4), respectively, were used to measure the activity and activity retention ratio of immobilized PGA.


(3) $${\text{EA}}_{\text{m}}=\frac{\text{C}\times \text{V}}{\text{m}\times \text{t}}$$

(4) $$\text{EAR}=\frac{\text{EAm}\times \text{m}}{\text{ELC}}\times 100\%$$

EA_m_ referred to the immobilization PGA activity (U/g), m denoted the quality of PGA that had been immobilized (g), which was the mass difference value of carriers before and after PGA was immobilized, and EAR denoted the activity retention ration of immobilization PGA (%), respectively.

## 3. Result and discussion

### 3.1. Determination of the optimum absorbance of glutaraldehyde

**Figure 1:**
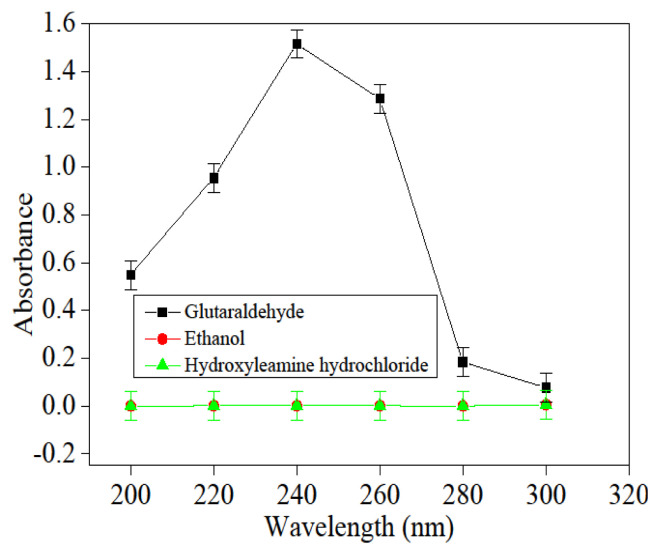
The relationship between absorbance and wavelength of glutaraldehyde.

A total of 1.000 mL of above-prepared glutaraldehyde solution was added with distilled water to 100.000 mL, and then 0.010 g/L glutaraldehyde solution was prepared. Transferred 5.00 mL of the above-obtained solution to a test tube that had been filled with 1.000 mL hydroxylamine hydrochloride with concentration 2.000 g/L, and shaking at 50 °C in an oscillator (Super Thermostatic Bath) for 10 min, the absorption of the solution was examined with a UV-752 N, Shanghai Precision Instrument Co. LTD) under various wavelengths using deionized water as a reference. The relationship between absorption (the even value of three determinations) and wavelength was displayed in Figure 1, the optimal absorbance spectrum was 240 nm. During this process, the hydroxylamine hydrochloride and ethanol absorbance were also tested in the same way to make sure the accuracy of the experiment, and the results were shown in Figure 1; it also was noted that ethanol and hydroxylamine hydrochloride were not absorbed at 240 nm, which meant that the concentration of glutaraldehyde could be accurately investigated.

### 3.2. Determination of glutaraldehyde reaction time

**Figure 2:**
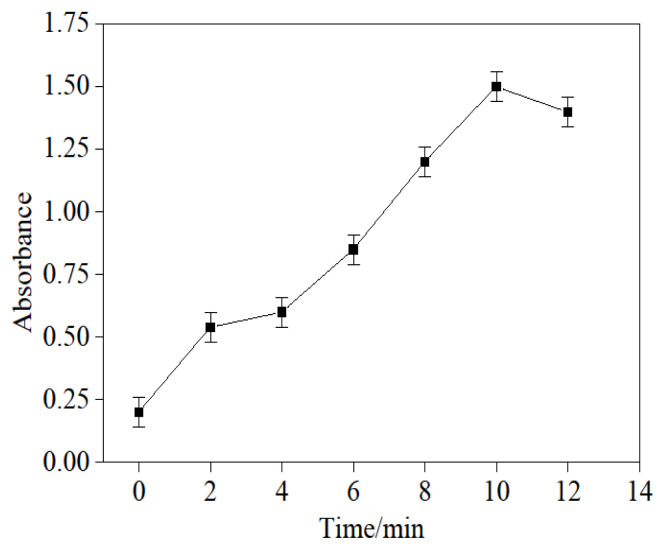
The relationship between oxime time and absorbance.

A total of 50.000 mL of 0.010 g/L glutaraldehyde solution was transferred to a flask, and then 10.000 mL hydroxylamine hydrochloride solution with a concentration of 2.000 g/L was introduced. The flask was placed into a 50 °C thermostatic water bath to react. After reacting for a given time, 5.50 mL of the solution was pipetted to measure; the absorbance was obtained at 240 nm using distilled water as a reference. In Figure 2, the relationship between reaction time and absorbance is shown, and, according to the result, the appropriate reaction time should be 10 min.

### 3.3. Preparation of glutaraldehyde standard curve

**Figure 3:**
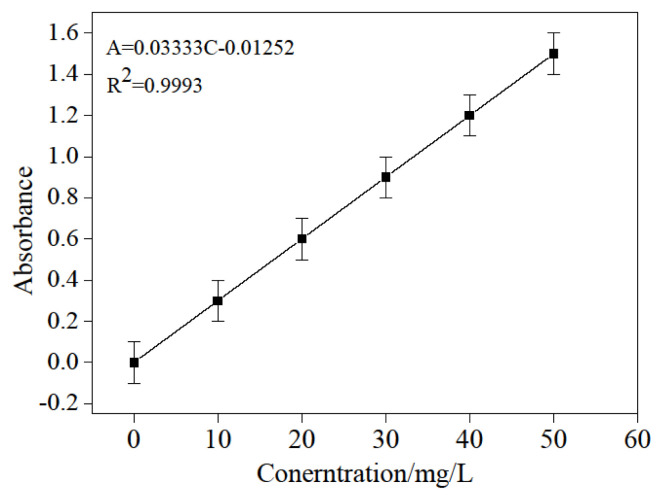
The relationship between absorption and concentration of glutaraldehyde.

1.00, 2.00, 3.00, 4.00, and 5.00 mL of 0.010 g/L above-prepared glutaraldehyde stock solution was used in a sequence of 100.000 mL of volumetric flasks; they were brought to scale with distilled water. After that, the relationship between absorbance (the even value of three determinations) and concentration was obtained using the same process carried out in “determination of maximum absorbance wavelength.” as shown in Figure 3. It presented a well linear relationship of A=0.03344C+0.00834(R^2^=0.9993) in range of concentration (0–50.0 mg/L).

### 3.4. The 6-APA optimum absorbance determination

**Figure 4:**
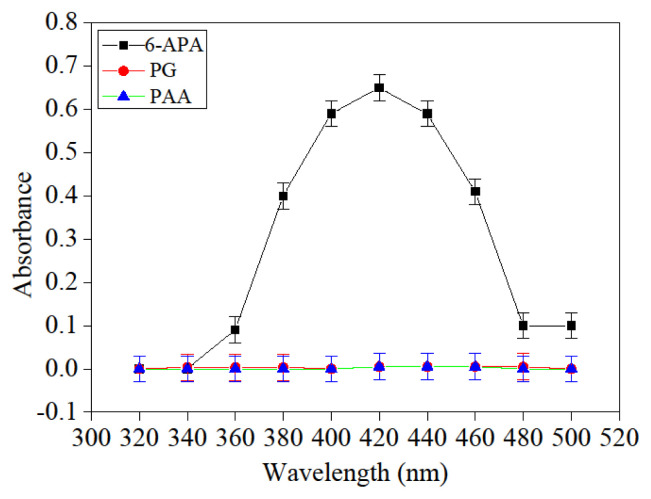
The relationship between absorbance and wavelength of 6-APA

A total of 0.500 mL of the above 6-APA solution with a concentration of 10.000 g/L was pipetted into a 10.000 mL colorimetric cylinder, and then 3.500 mL of the above prepared PDAB solution was introduced. After the solution was reacted at an ambient temperature for 3 min, the absorbance was determined at different wavelengths three times on the UV-752 N; the relationship between absorption (the even value of three determinations) and wavelength was demonstrated in Figure 4. It could be observed that the optimum absorption was displayed at 420 nm. Simultaneously, the absorption of PAA and PG at different wavelengths was measured in the same method as 6-APA, except 6-APA that was not introduced, and the result indicated that there was no absorption at 420 nm for PAA and PG, which showed that the 6-APA concentration could be determined accurately at 420 nm.

### 3.5. The 6-APA standard curve preparation

**Figure 5:**
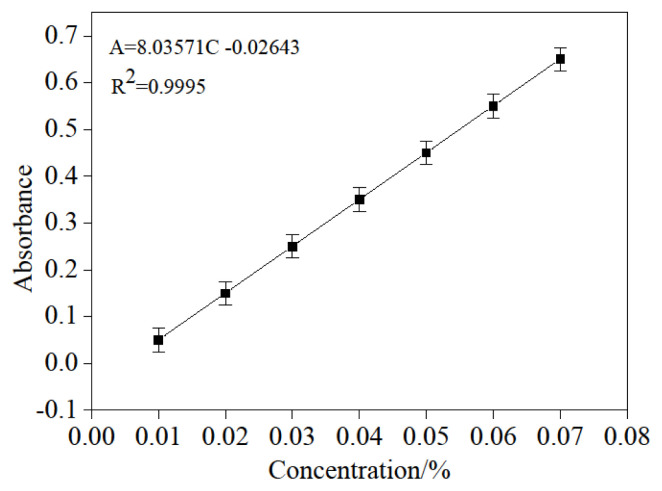
The relationship between absorbance and concentration of 6-APA.

2.500, 5.000, 7.500, 10.000, 12.500, 15.000, 17.500 mL of the 10.000 g/L standard 6-APA solution was put into a sequence of 50.000 mL volumetric flasks, and then they were brought to scale again, and a series of the standard solution was obtained. Afterward, the chromogenic reaction was performed in the same process as illustrated in “maximum absorbance determination of 6-APA”; the solution absorption was measured at 420 nm three times. The relationship between the absorbance (the even value of three determinations) and the concentration was achieved in Figure 5. It presented a well linear relationship of A=8.0357C-0.0264(R^2^=0.9995) in the range of concentration (0~0.0756) g/L.

### 3.6. Characterization of the materials

#### 3.6.1. FTIR Characterization of Fe_3_O_4_@SiO_2_-NH_2_ nano-magnetic composite and Fe_3_O_4_@SiO_2_-NH_2_-Glu-PGA

**Figure 6:**
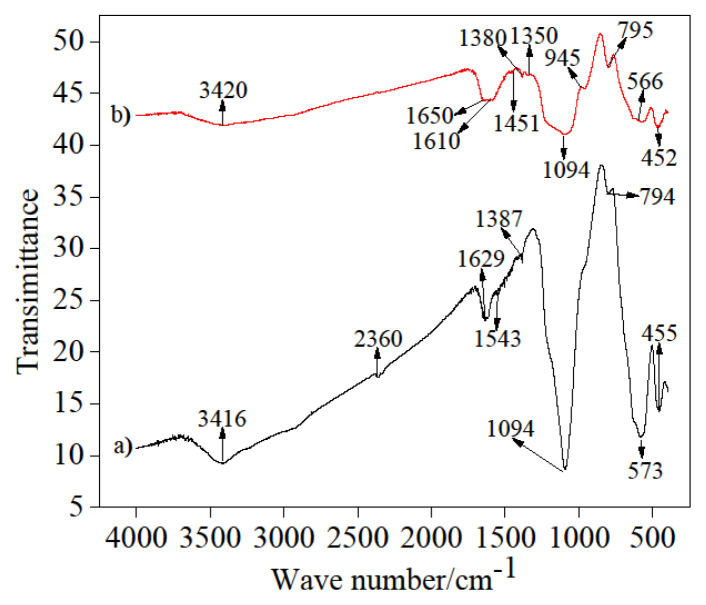
FTIR spectra of (a) Fe_3_O_4_@SiO_2_-NH_2_ nano-magnetic composite_,_ and (b) Fe_3_O_4_@SiO_2_-NH_2_-Glu-PGA.

After drying magnetic nanoparticle and its composites in a vacuum oven for 48 h at 40 °C, 1–2 mg from product was weighed and mixed with KBr in 1–100 (w/w) ratio; the mixture tablet, and sample cakes were obtained. The wavenumber scanning sample was in the range of 400–4000 cm^−1^, which was observed by an FTIR (IFS66V/S, Bruker, Germany); all peaks were recorded. Figure 6a shows the infrared spectra of Fe_3_O_4_@SiO_2_-NH_2_ nano-magnetic composite, with the peak at 3416 cm^−1^, which could be explained to stretching vibration OH of Fe_3_O_4_@SiO_2_-NH_2_ nano-magnetic composite, and the presence of H_2_O existed on it is surface; the peaks at 573, 455 cm^−1^ were due to the existence of stretching Fe-O. Asymmetric and symmetrical stretching vibrations of Si-O-Si bonds in oxygen-silica tetrahedrons were observed at 1094 cm^−1^; the peak at 1629 cm^−1^ was explained to the free NH_2_ stretching vibration. The spectra is shown in Figure 6b. Fe_3_O_4_@SiO_2_-NH_2_-Glu-PGA has verified the coating of silica on magnetic nanoparticles. The peak at 566 cm^−1^ was indicated by the Fe-O bond. In Fe_3_O_4_@SiO_2_-NH_2_-Glu-PGA spectra, asymmetric and symmetrical stretching vibrations of Si-O-Si bonds in oxygen-silica tetrahedrons were observed at 1094 and 452 cm^−1^. The peak at 1650 cm^−1^ was illustrated C=N, -CH=N- of PGA. The peaks at 795, 945 cm^−1^, respectively, were represented to NH_2_ of APTES stretching vibration on the surface of the particles after decorating was successfully applied. The peaks 1380 and 1350 cm^−1^ were referred to as CH’s and CH_2_’s stretching vibration of glutaraldehyde, respectively. And also, the peak at 1451cm^−1^ was allocated to C=O of glutaraldehyde in Fe_3_O_4_@SiO_2_-NH_2_-Glu-PGA of PGA. The broad characteristic band at 3420 cm^−1^ is denoted to OH stretching vibration of Fe_3_O_4_ and NH stretching vibration of PGA.

#### 3.6.2. XRD studies of Fe_3_O_4_ magnetic nano-particles and Fe_3_O_4_@SiO_2_-NH_2_-Glu-PGA

**Figure 7:**
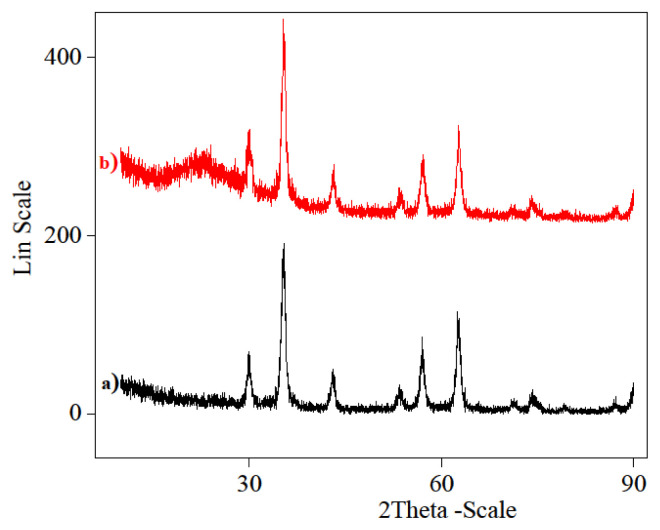
XRD pattern of (a) Fe_3_O_4_ magnetic nanoparticles and (b)Fe_3_O4@SiO_2_-NH_2_-Glu-PGA.

XRD (Rigaku d/max-2400 X-ray) spectroscopy had been applied to study the structure of Fe_3_O_4_ and Fe_3_O_4_ @SiO_2_-NH_2_-Glu-PGA (Figure 7). The naked Fe_3_O_4_ nano-magnetic particles XRD diagram (Figure 7a) showed patterns that included sequence spinel ferrites, and peaks showed up at 2θ = 30.02°, 35.38°, 43.10 °, 53.45°, 57.06°, and 62.62° were related to the high cubic crystalline structure of Fe_3_O_4_ nano-magnetic particles. The same groups of characteristic peaks position were identified in Fe_3_O_4_@SiO_2_-NH_2_-Glu-PGA (Figure 7b), demonstrating the stabilization of crystal structure of Fe_3_O_4_ nano-magnetic particles throughout silica coating and amino surface functionality. In the Fe_3_O_4_@SiO_2_-NH_2_-Glu-PGA nano-magnetic composites, the amorphous SiO_2_ layer showed a wide signal between 15° and 30° and the result was in consistency with several authors [49]. Furthermore, XRD results illustrated that the influence of the modification on the core-shell crystal sample composition was negligible. After modification, a decrease in peak intensity was specifically explained with the silica shells coated on the particles surface [50].

#### 3.6.3. Magnetic properties of Fe_3_O_4_ magnetic nano-particles and Fe_3_O_4_@SiO_2_-NH_2_-Glu-PGA

**Figure 8:**
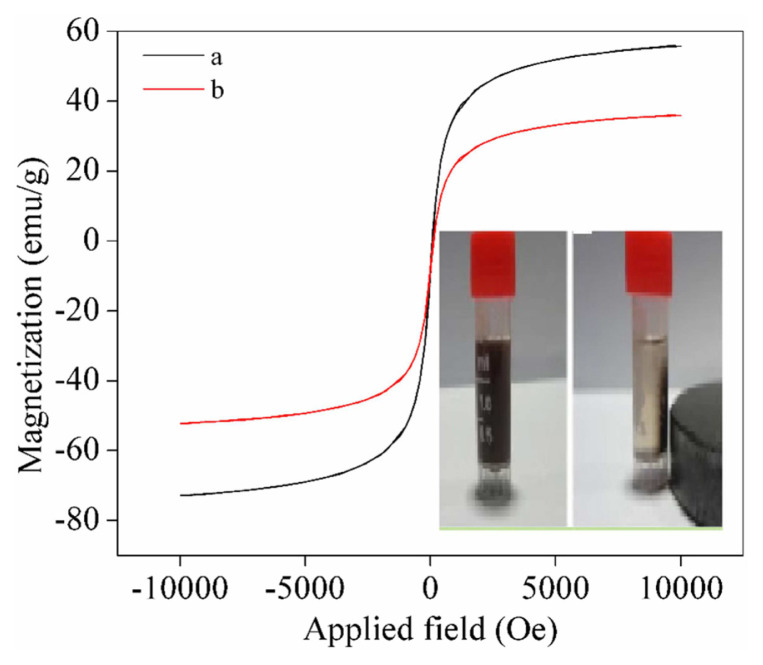
Magnetic hysteresis curve of (a) Fe_3_O_4_ magnetic nanoparticles, and (b)Fe_3_O_4_@SiO_2_-NH_2_-Glu-PGA.

The VSM (MPMS-SQUID VSM-094) was applied to measure the magnetic properties and the field dependency hysteresis loops (M-H curves) of Fe_3_O_4_ and Fe_3_O_4_@SiO_2_-NH_2_-Glu-PGA. No decreased remanence and coerciveness were observed, confirming that Fe_3_O_4_ and Fe_3_O_4_@SiO_2_-NH_2_-Glu-PGA were superparamagnetic at room temperature. Fe_3_O_4_ and Fe_3_O_4_@SiO_2_-NH_2_-Glu-PGA saturation magnetizations were 55 and 35 emu/g, respectively, resulting from the later modification of Fe_3_O_4_ and the immobilization of PGA, both of which would decrease the relative content of Fe_3_O_4_. As seen in Figure 8, as the magnet was fixed on one of the bottle sides, the microparticles were collected on the bottle wall near the magnet, and the solution remained visible in short period of the time. The prepared MNPs were quickly dispersed by careful shaking in the solution as soon as the magnet was removed as seen in Figure 8. The foundings demonstrated that the prepared Fe_3_O_4_@SiO_2_-NH_2_-Glu-PGA had outstanding magnetic properties, enabling it to be used in the target delivery field.

#### 3.6.4. SEM-EDS characterization of Fe_3_O_4_ nano-magnetic particles Fe_3_O_4_@SiO_2_-NH_2_-Glu-PGA

**Figure 9a:**
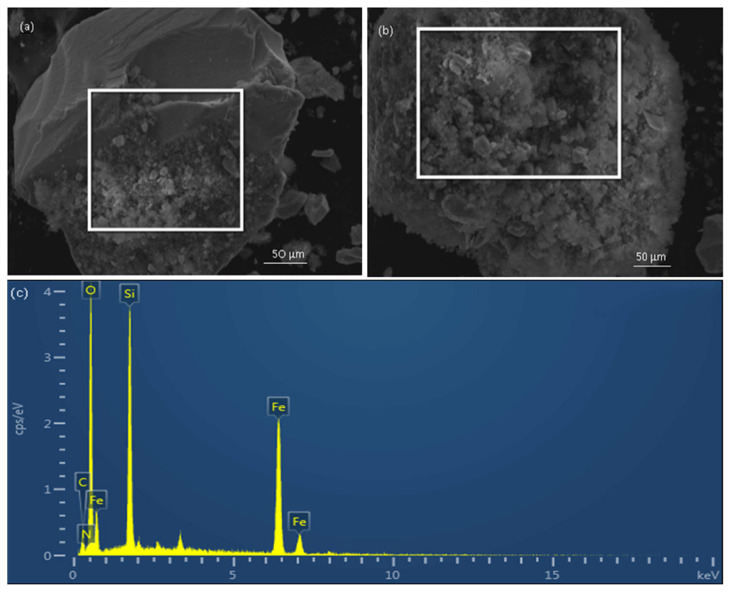
SEM-EDS micrograph of Fe_3_O_4_ magnetic nanoparticles. Figure 9b. SEM-EDS micrograph of Fe_3_O_4_@SiO_2_-NH_2_-Glu-PGA. Figure 9c. SEM-EDS spectrum of Fe_3_O_4_ @SiO_2_-NH_2_-Glu-PGA.

Fe_3_O_4_ (Figure 9a) and Fe_3_O_4_@SiO_2_-NH_2_-Glu-PGA (Figure 9b) micrographs of morphologies were obtained to use an electron scanning microscope, with an extremely high voltage configuration of 15 kV. The photographs were explained in Figure 9a, the Fe_3_O_4_ was in shape of nearly spherical particles with a diameter of 16.5 nm. In Figure 9b, the particles were bigger after immobilization and appear to be aggregated, which could be related to the reaction that occurred on surface of Fe_3_O_4_@SiO_2_-NH_2_-Glu-PGA. In Figure 9c, SEM-EDS (JEOL JSM-5800V) analysis was also used for the assessment of chemical purity and elementary composition. The existence of Fe, Si, and O were confirmed by the SEM-EDS spectrum.

### 3.7. Free and immobilized PGA thermal stability

**Figure 10:**
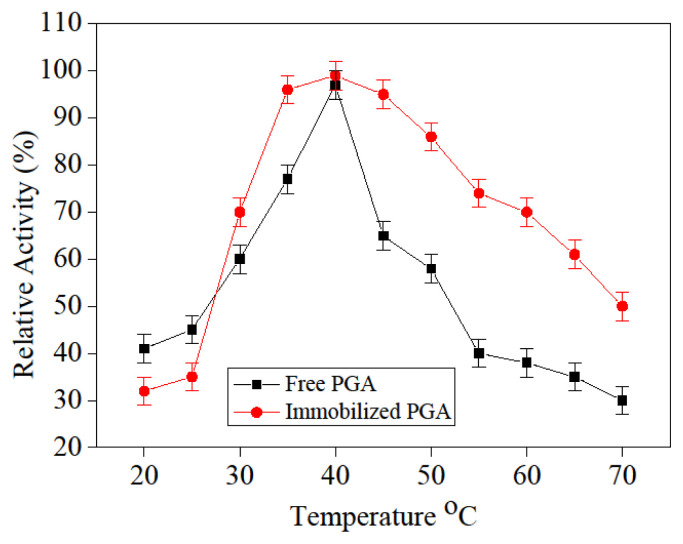
Influence of thermal stability on free and immobilized PGA.

The temperature influence on the free and immobilization PGA relative activity was displayed in Figure 10. Immobilized PGA demonstrated important and significant variation in the pattern of temperature behavior with that of free ones in the temperature range studied (20–70 °C). The maximum activity was measured at 40 °C for free and immobilization PGA, and immobilization PGA typically exhibited greater activity than free PGA, indicating the highest thermal stability. The raise in ideal temperatures were promoted by an increase in immobilized PGA rigidity. The functional group of enzymes on nano-magnetic composites had been in significantly limited interaction with each other. Increased stabilization of the immobilization PGA may be caused by reduced autolysis. The temperature vaşue of 40 °C was the critical temperature for the free PGA since the activity retained at 60% after that temperature. Eventually, at 70 °C, the loss of enzyme activity was estimated to be 30% for immobilized PGA and 65% for free.

### 3.8. Free and immobilized PGA pH stability

**Figure 11:**
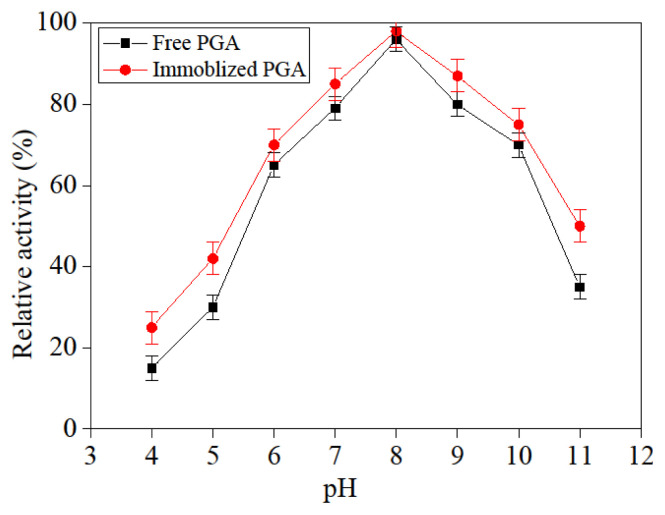
Influence of pH on free and immobilized PGA.

The pH value of the reaction medium would influence catalytic activity. The free and immobilization PGA activity, under various pH values, was shown in Figure 11; it presented that free PGA and immobilized PGA displayed the maximum activity at pH = 8. But the pH stability of immobilization PGA had been greatly improved PGA significantly. As result, the nanoparticle used did not substantially alter the chemical environment of immobilization PGA when compared to the free PGA, undoubtedly, the solidity of the immobilized PGA increased the activity of the PGA in various pH values compared to the free PGA.

### 3.9. Time stability influence of free and immobilized PGA

**Figure 12:**
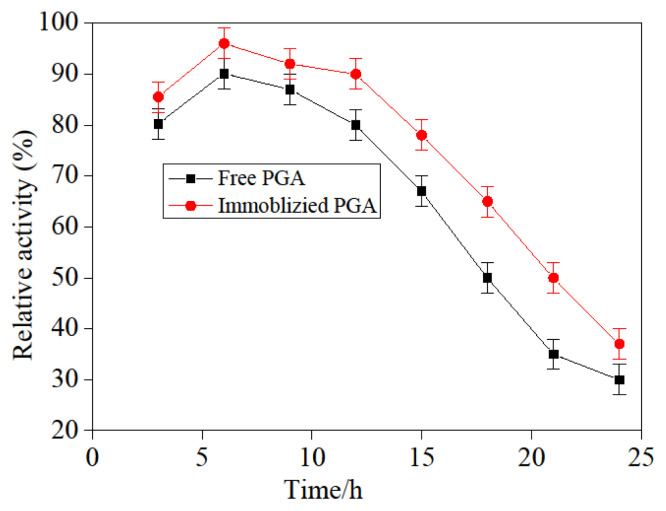
Influence of time stability on free and immobilized PGA.

The time stabilization influences on free and immobilization PGA were illustrated in Figure 12, the activity of both enzymes was enhanced firstly and decreased with time, and the maximal activity was seen at the 6th h with the free and immobilized PGA time. The explanations for this phenomenon were as follows: the PGA was not enough to be covalently attached to the carrier when the immobilization time had been 3 h, so the free and immobilization PGA activity was smaller; although the free and immobilization PGA activity peaked at 6 h, it means the enzymes’ activity rate was relatively higher. Moreover, the free and immobilized PGA activity gradually declined.

### 3.10. Immobilized PGA reusability

**Figure 13:**
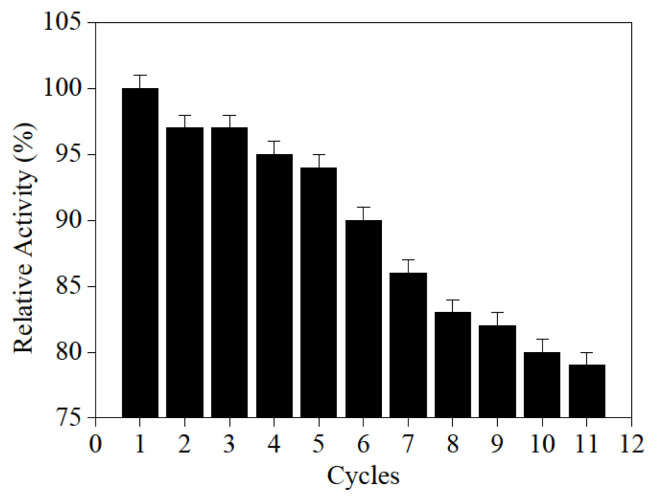
The reusability of immobilized PGA.

The recycling property is the most essential and appealing property of the immobilized enzyme. The reproducibility of the catalyst was estimated by immobilization PGA activity as a result of the reuse number of immobilization PGA in the (50 mM, pH 7.8) phosphate buffer solution in the assay. Figure 13 showed the residual activity of PGA immobilization during reuse. After recycling for 11 cycles, the immobilized PGA retained approximately 79% of it, which revealed good reusability. PGA immobilization on surface-modified magnetic Fe_3_O_4_@SiO_2_-NH_2_-Glu-PGA provides new support for excellent enzyme catalyst immobilization and sufficient stability and reuse of nano-magnetic composite immobilized PGA.

### 3.11. The advantages of this work

To determine the value of our study, we compared data from our experiment with data from PGA immobilized on different carriers, and the findings were given in Table. It was demonstrated that immobilized PGA on a carrier of Glu modified Fe_3_O_4_@SiO_2_-NH_2_ had excellent catalytic activity. Furthermore, the catalytic decreasing ratio was lower, which could be explained by the carrier’s suitable bonding efficiency and environment. The results of the experiments indicated that glutaraldehyde could not only effectively enhance its stability but also contain the catalytic activity at a high level [54, 55], and higher catalytic activity could be easily comprehended. Moreover, the interaction between glutaraldehyde molecules and primary amino groups was significantly simpler than that of others [56], implying that the secondary reaction that occurs on immobilized PGA could be avoided at a wide scale. Besides that, there was still a lot of hydroxyls on the surface of the modified nano-magnetic composite, and our experiments revealed that if PGA was surrounded by hydroxyl, a high activity could be achieved.

## 4. Conclusion

In this study, the magnetite nanoparticles were co-precipitated, and the silica-coated magnetic particles were then synthesized using the Stöber process. To obtain NH_2_-modified nano-magnetic particles, the silica-coated Fe_3_O_4_ nano-magnetic particles were treated with APTES, and penicillin G acylase was covalently immobilized. Glutaraldehyde was the functionalized nanomagnetic composites to attach penicillin G acylase molecules. The FTIR spectrum has been used to confirm nano-magnetic particles and the Penicillin G acylase immobilization. The XRD pattern revealed that Fe_3_O_4_ phase change was not the result of the immobilization operation. The SEM-EDS images demonstrated the magnetite nanoparticles diameter were approximately 35 nm, while the diameter of PGA magnetite nanocomposites was 100nm, and the SEM-EDX analysis also showed that the magnetite nanoparticles had already been successfully coated with silica. The effect of immobilization conditions on the catalytic performance of immobilization PGA has been investigated. The optimum immobilization reaction occurred at an immobilized temperature of 40 °C, pH = 8.0, and the immobilization period of 6 h. The findings for ELC was 9198 U, for EA was 14602 U/g, and for EAR was 87.7% under optimal process conditions. Ultimately, the results showed that thermal stability, pH stability, and reaction time stability of immobilized PGA were increased dramatically to free PGA, after 11 repeated cycles of operation immobilized PGA remained at 79%.

## Figures and Tables

**Table:** Summary of activities of PGA immobilized on different support materials.

Support	ELC	EA	EAR	Ref.
Sio_2_-Glu	9190 U	14969 U/g	88.5%	[38]
Fe_3_O_4_@PDA-Glu	114 mg/g	26,308 U/g	78.5%	[51]
Fe_3_O_4_@3(trimethoxysilyl) propylmethacrylate@GMA	64.08%	9208 U/g	88 mg/g	[52]
TiO_2_@3-GCDPTMS	10800 U	14900 U/g	30%	[53]
Fe_3_O_4_@SiO_2_-NH_2_-Glu	9198 U	14602 U/g	87.7%	This work

Naturally comprehended despite the fact that if PGA was immobilized on Fe_3_O_4_@SiO_2_-NH_2_, the interaction between PGA and carrier would be a covalent bond, the immobilization PGA could not drop off from the carrier under normal operation circumstances, and it maintained excellent stability.
